# Mechanism of Adenovirus E4-ORF3-Mediated SUMO Modifications

**DOI:** 10.1128/mBio.00022-19

**Published:** 2019-02-26

**Authors:** Sook-Young Sohn, Patrick Hearing

**Affiliations:** a Department of Molecular Genetics and Microbiology, School of Medicine, Stony Brook University, Stony Brook, New York, USA; University of Michigan-Ann Arbor; University of Colorado at Boulder; Tufts University School of Medicine

**Keywords:** adenoviruses, E3 ligase, E4-ORF3, SUMO, UBC9

## Abstract

Regulation of a variety of different cellular processes, including posttranslational modifications, is critical for the ability of many viruses to replicate efficiently within host cells. The adenovirus (Ad) E4-ORF3 protein assembles into polymers and forms a unique nuclear scaffold that leads to the relocalization and sequestration of cellular proteins, including small ubiquitin-like modifiers (SUMOs). Previously, we showed that E4-ORF3 functions as a SUMO E3 ligase of transcriptional intermediary factor-1 gamma (TIF-1γ) and promotes poly-SUMO chain formation. Here, we present cellular and biochemical data to further understand E4-ORF3 SUMO ligase activity. E4-ORF3 proteins from five different Ad species were found to possess SUMO E3 ligase activities *in vitro*. In infected cells, SUMO modifications of target proteins occurred only when the proteins were recruited into E4-ORF3 polymeric structures. By analyzing SUMO-deficient TIF-1γ, we demonstrated that SUMO conjugations are not required for E4-ORF3-mediated relocalization of target proteins in infected cells, implying that sequestration is followed by SUMO modification. *In vitro* SUMO conjugation assays revealed the Ad E1B-55K oncoprotein as a new viral target of E4-ORF3-mediated SUMOylation. We also verified a direct function of E4-ORF3 as a SUMO ligase for multiple cellular proteins, including transcription factor II-I (TFII-I), Nbs1, and Mre11. Moreover, we discovered that E4-ORF3 associates with SUMO-bound UBC9, and E4-ORF3 polymerization is crucial for this ternary interaction. Together, our findings characterize E4-ORF3 as a novel polymer-type SUMO E3 ligase and provide mechanistic insights into the role of E4-ORF3 in SUMO conjugation.

## Abstract (precis)

Viruses interplay with the host SUMOylation system to manipulate diverse cellular responses. The Ad E4-ORF3 protein forms a dynamic nuclear network to interfere with and exploit different host processes, including the DNA damage and interferon responses. We previously reported that E4-ORF3 is a SUMO E3 ligase. Here, we demonstrate that this activity is a conserved function of evolutionarily diverse human Ad E4-ORF3 proteins and that E4-ORF3 functions directly to promote SUMO conjugations to multiple cellular proteins. Recruitment of cellular substrates into E4-ORF3 nuclear inclusions is required for SUMO conjugation to occur *in vivo*. We probed the mechanism by which E4-ORF3 functions as a SUMO E3 ligase. Only multimeric, but not dimeric, E4-ORF3 binds to the SUMO E2 conjugation enzyme UBC9 *in vitro* only in a trimeric complex with SUMO. These results reveal a novel mechanism by which a conserved viral protein usurps the cellular SUMO conjugation machinery.

## INTRODUCTION

The DNA tumor virus adenovirus (Ad) has evolved different mechanisms to target host cell processes to optimize the cellular environment during infection. Studies of the Ad replication cycle have revealed fundamental insights into the regulation of gene expression, protein translation, cell proliferation, and cell death (1). Studies of Ad infection have also provided unique insights into innate host responses to viral infection, including the DNA damage response (DDR) and an interferon (IFN) response (2, 3). The linear double-stranded Ad genome is sensed in the cytoplasm to stimulate type I IFN production (2). Nuclear Ad DNA is sensed as double-strand breaks to trigger a DDR (3). The DDR and IFN responses significantly inhibit Ad replication if left unabated. Consequently, Ad has evolved multiple mechanisms to interfere with these innate immune pathways.

The Ad E4-ORF3 protein functions by relocalizing and sequestering cellular proteins into filamentous nuclear inclusions (4, 5). To prevent a DDR, E4-ORF3 sequesters Mre11, Rad50, and Nbs1 (the MRN complex) (6–8), which functions as a DNA damage sensor (9). With IFN signaling, E4-ORF3 relocalizes effectors of the pathway, including promyelocytic leukemia (PML) protein and PML nuclear body (PML-NB) components, including Sp100 and Daxx (4, 5, 10, 11). The E4-ORF3 protein also targets several transcription factors, including tripartite motif containing protein 24 (TRIM24)/transcriptional intermediary factor-1 alpha (TIF-1α), TRIM33/TIF-1γ, and transcription factor II-I (TFII-I) (1, 12–15). Ads are ubiquitous pathogens that infect a wide range of vertebrates. Nearly 90 human Ad types have been identified that are grouped into eight species (A to H). Interestingly, the E4-ORF3 proteins from different Ad species all target PML-NBs for disruption, but only species C Ad E4-ORF3 relocalizes MRN complex proteins (7).

Many cellular proteins that are relocalized by E4-ORF3 are modified by the small ubiquitin-like modifier (SUMO) (16, 17). SUMO modification regulates diverse cellular processes, including transcription, replication, DNA repair, protein localization, and protein stability, mostly by modulating protein-protein interactions (18, 19). SUMO conjugation to target lysine residues in substrates follows the paradigm of the ubiquitin conjugation system (18, 19). The C-terminal diglycine motif of a processed SUMO is conjugated to a catalytic cysteine residue of the E1-activating enzyme. SUMO is then transferred to the catalytic cysteine of the E2-conjugating enzyme. In humans, there is one heterodimeric SUMO E1-activating enzyme (SAE1/SAE2) and one E2-conjugating enzyme (UBC9). SUMO may be directly transferred from the E2 enzyme to a substrate or the activity of an E3 ligase may promote the process. Only a limited number of SUMO E3 ligases have been identified, and much less is known about them compared with ubiquitin E3 ligases. In humans, three SUMO paralogs, SUMO-1, -2, and -3 are best characterized in this family (18, 19). Mature SUMO-2 and SUMO-3 proteins are 97% identical and often referred to as SUMO-2/3. The SUMO-1 protein is ∼50% identical to SUMO-2/3. SUMO-2/3, but not SUMO-1, can form polymeric SUMO chains via conjugation at SUMO-2/3 lysine residue 11.

We previously demonstrated that the Ad5 E4-ORF3 protein promotes SUMO modifications of multiple cellular proteins and further targets some of them for ubiquitin-dependent proteasomal degradation (1, 12, 16, 17, 20). Using *in vitro* SUMOylation assays, we showed that the Ad5 E4-ORF3 protein itself functions as a SUMO E3 ligase (20). These findings place the Ad E4-ORF3 protein at the nexus of the cellular SUMOylation system. A growing number of studies have demonstrated that viruses manipulate host SUMOylation pathways (21). Interestingly, it was found that disruption of global SUMO modification in immune cells results in enhanced type I IFN responses (22, 23). This result suggests that SUMOylation plays important roles in aspects of antiviral activity as well as virus replication.

In this report, we further characterized E4-ORF3-mediated SUMOylation by analyzing E4-ORF3 proteins from evolutionarily diverse human Ads and if divergent E4-ORF3 target proteins are regulated by distinct mechanisms. Our results demonstrate that *in vitro* SUMO ligase activity is conserved across human Ad types, and that recruitment of cellular proteins into E4-ORF3 nuclear inclusions is required for their SUMOylation in infected cells. Using *in vitro* binding assays, we reveal a novel mechanism by which the E4-ORF3 protein interacts with E2 UBC9 and SUMO-3 via formation of a trimeric protein complex. Overall, these results provide mechanistic insights into the activity of a conserved viral protein whose function impacts multiple nuclear signaling pathways by a unique mechanism.

## RESULTS

### Ad E4-ORF3-mediated TIF-1γ subnuclear relocalization and E4-ORF3 SUMO E3 ligase activities are conserved among human Ad types.

**FIG 1:**
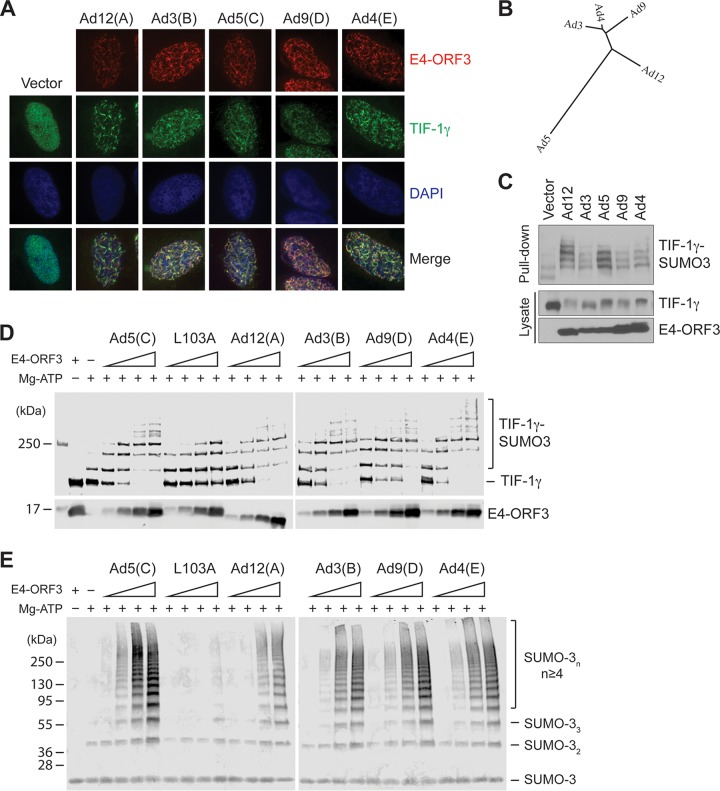
Ad E4-ORF3-mediated TIF-1γ subnuclear relocalization and E4-ORF3 SUMO E3 ligase activities are conserved among human Ad types. HeLa (A) or His_6_-tagged SUMO3-expressing HeLa (C) cells were infected with 500 particles/cell (p/cell) of recombinant empty AdCMV (Vector) or AdCMV-HA-E4-ORF3 from Ad12, 3, 5, 9, and 4 belonging to species A, B, C, D, and E, respectively. (A) At 8 h postinfection (hpi), E4-ORF3 and TIF-1γ were immunostained with anti-HA and anti-TIF-1γ antibodies and visualized by fluorescence microscopy. (B) The phylogenetic relationships of E4-ORF3 proteins. (C) The phylogenetic tree was created using the Phylogeny.Fr server (43). SUMO-conjugated TIF-1γ (Pull-down) was captured by Ni-NTA agarose beads and analyzed by Western blotting. (D) GST-TIF-1γΔC (100 nM) was incubated with 50 nM E1, 250 nM E2, 50 μM His_6_-SUMO-3, and increasing concentrations of His_6_-E4-ORF3 proteins (0.33, 1, 3, and 9 μM) at 37°C for 60 min. Reaction mixtures were analyzed by Western blotting with anti-TIF-1γ and anti-His antibodies. (E) Increasing concentrations of His_6_-E4-ORF3 proteins (0.17, 0.5, 1.5, and 4.5 μM) were added to E1/E2/SUMO-3 reaction mixtures described for panel D and incubated at 37°C for 60 min. Products were analyzed by Western blotting with the anti-SUMO-2/3 antibody. DAPI, 4′,6-diamidino-2-phenylindole.

Ad E4-ORF3 target proteins can be divided into two groups based on their altered subnuclear localization mediated by different human Ad types. One group of targets, such as Mre11, Nbs1, Rad50, and TFII-I, are sequestered by E4-ORF3 from only species C Ads, such as Ad2 and Ad5 (7, 12, 17). On the other hand, PML, TIF-1α, and SUMO-1, -2, and -3 are recruited into nuclear inclusions formed by E4-ORF3 from all types of Ads examined (7, 14, 17). E4-ORF3 proteins from Ad5 and Ad12 relocalize TIF-1γ into nuclear inclusions (13, 15, 20). We determined the effect of E4-ORF3 from Ad species A to E on TIF-1γ localization by immunofluorescence. HeLa cells were infected with recombinant Ads expressing hemagglutinin (HA)-tagged E4-ORF3 proteins from Ad12, 3, 5, 9, and 4 belonging to species A, B, C, D, and E, respectively. Since E4-ORF3 induces proteasomal degradation of TIF-1γ (15, 20), we examined TIF-1γ localization in Ad-infected cells before a major loss of protein takes place (8 hours postinfection [hpi]). As seen in Fig. 1A, TIF-1γ was relocalized by the E4-ORF3 proteins from all five different species.

Our previous study revealed that Ad5 E4-ORF3 functions as a SUMO E3 ligase for TIF-1γ using an *in vitro* SUMOylation reaction (20). We also showed that Ad5 E4-ORF3 promotes poly-SUMO-3 chain formation in the absence of substrate. Phylogenetic relationships of the E4-ORF3 proteins of different Ad types are shown in Fig. 1B. The Ad5 E4-ORF3 protein has diverged significantly from those of other Ad species. Therefore, we investigated whether this SUMO E3 ligase activity of E4-ORF3 is conserved among the Ad types. In cultured cells, all the E4-ORF3 proteins tested were able to enhance SUMO-3 conjugation to TIF-1γ and promote TIF-1γ degradation (Fig. 1C, pulldown and lysate, respectively). We next analyzed SUMO E3 ligase activities using an *in vitro* SUMO conjugation assay. Recombinant E4-ORF3 proteins were purified from Escherichia coli and their ligase activities determined in the absence or presence of a substrate, glutathione *S*-transferase (GST)-tagged TIF-1γΔCT (20). As described in our previous report (20), we optimized the reaction conditions such that weak TIF-1γ SUMOylation was evident with E2 alone. We then incubated reaction mixtures with increasing concentrations of E4-ORF3 proteins. Consistent with the observation in Fig. 1B, the E4-ORF3 proteins of all different Ad types facilitated SUMO-3 conjugation to TIF-1γ (Fig. 1D), as well as poly-SUMO-3 chain formation (Fig. 1E). The Ad5 E4-ORF3 L103A mutant, which is defective in the formation of polymeric nuclear tracks and subsequent E4-ORF3 functions (6), was used as a negative control. Taken together, our data indicate that SUMO E3 ligase activities of E4-ORF3 for TIF-1γ SUMOylation and poly-SUMO chain formation are conserved across human Ad species.

### E4-ORF3 enhances SUMO-1 and SUMO-3 conjugation to TIF-1γ and TFII-I.

**FIG 2:**
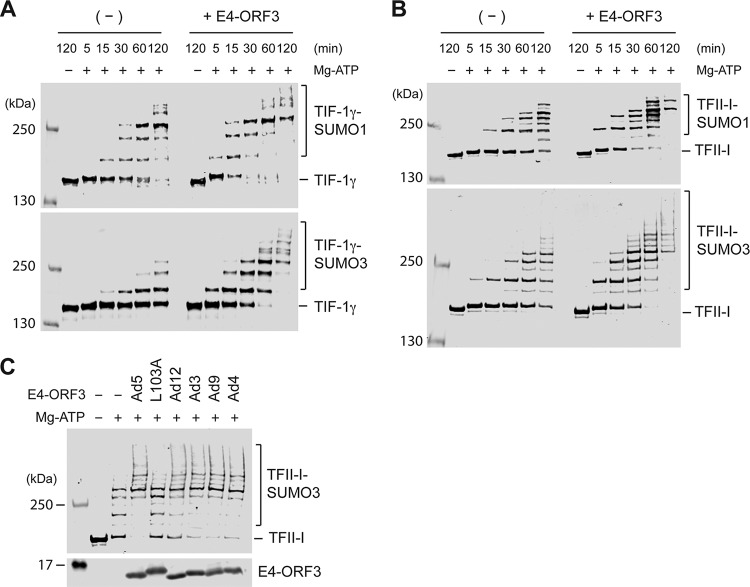
SUMO-1 and SUMO-3 conjugations to TIF-1γ and TFII-I. (A and B) GST-TIF-1γΔC (A) or GST-TFII-I (B) was incubated in E1/E2 and SUMO-1 (top) or SUMO-3 (bottom) reaction mixtures with (*+ E4-*ORF3) or without (*−*) Ad5 E4-ORF3 (3 μM) for the indicated time periods. Proteins were detected with anti-TIF-1γ and anti-TFII-I antibodies. (C) GST-TFII-I was incubated in reaction mixtures described in Fig. 1D with 3 μM His_6_-E4-ORF3 proteins and incubated at 37°C for 60 min. Products were analyzed by Western blotting with anti-TFII-I and anti-His antibodies.

We previously found that Ad5 E4-ORF3 induces SUMO conjugation to TFII-I in cultured cells (12, 16). To determine whether E4-ORF3 directly functions as a SUMO E3 ligase for TFII-I, as it does to TIF-1γ, TFII-I protein was purified from E. coli (GST-TFII-I) and analyzed as a substrate *in vitro* for SUMO-3 conjugation in the absence or presence of E4-ORF3. Similar to TIF-1γ (Fig. 2A, bottom), TFII-I was modified by SUMO-3 with E2 alone, and this modification was significantly enhanced by incubation with Ad5 E4-ORF3 (Fig. 2B, bottom, – versus + E4-ORF3). This result demonstrates that Ad5 E4-ORF3 directly acts as a SUMO E3 ligase for TFII-I. In cultured cells, E4-ORF3 induces both SUMO-1 and SUMO-3 modification of TFII-I (12). Thus, we sought to determine whether E4-ORF3 itself can induce SUMO-1 conjugation to TIF-1γ and TFII-I *in vitro*. Interestingly, E4-ORF3 exhibited E3 ligase activity for SUMO-1 conjugation to these substrates (Fig. 2A and B, top).

TFII-I subnuclear relocalization and SUMOylation are induced by E4-ORF3 from only species C (Ad5) but not by any other Ad types in cultured cells (12). Because E4-ORF3 proteins from five different types showed E3 ligase activities for TIF-1γ SUMOylation and poly-SUMO-3 chain formation (Fig. 1D and E), we asked whether the ligase activity of E4-ORF3 for TFII-I SUMOylation is specific for Ad5 *in vitro*. We performed an *in vitro* SUMOylation assay of TFII-I using E4-ORF3 proteins from five types and compared SUMOylation levels. All of the E4-ORF3 proteins were able to promote SUMO-3 conjugation to TFII-I *in vitro* (Fig. 2C), indicating that E4-ORF3 proteins from different Ad types possess E3 ligase activities for TFII-I SUMOylation. These results imply that the defect in induction of TFII-I SUMOylation displayed by E4-ORF3 proteins from Ad types other than species C *in vivo* is mainly caused by a lack of their ability to relocalize TFII-I into E4-ORF3 nuclear inclusions, not by a lack of E4-ORF3 SUMO E3 ligase activities.

### Ad5 E4-ORF3 functions as a SUMO E3 ligase for Nbs1, Mre11, and Ad5 E1B-55K.

**FIG 3:**
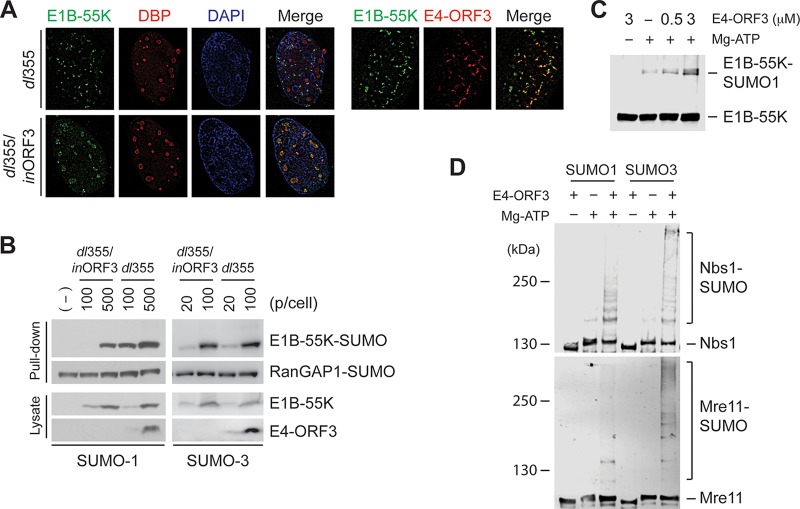
E4-ORF3 induces SUMO conjugation to E1B-55K, Nbs1, and Mre11. (A) HeLa cells infected with *dl*355 (ΔE4-ORF6) or *dl*355-*in*ORF3 (ΔE4-ORF3/ΔE4-ORF6) were immunostained for E1B-55K and E2-DBP or E4-ORF3 at 12 hpi. (B) His_6_-tagged SUMO-1 or SUMO-3-expressing HeLa cells were infected with indicating amounts of *dl*355 or *dl*355-*in*ORF3. At 12 hpi, SUMO conjugates were captured by Ni-NTA agarose beads and analyzed by Western blotting with the anti-E1B-55K antibody. SUMO-conjugated RanGAP1 was shown as a control for SUMO capture. Protein levels of E1B-55K and E4-ORF3 from cell lysates were determined by Western blotting. (C) GST-tagged Ad5 E1B-55K was incubated with SUMO-1/E1/E2 and the indicated concentrations of Ad5 E4-ORF3. The levels of SUMO conjugation were analyzed by Western blotting with the anti-E1B-55K antibody. (D) GST-tagged Nbs1 and Mre11 were incubated with SUMO E1/E2 and SUMO-1 or SUMO-3 in the absence or presence of Ad5 E4-ORF3 (3 μM). The reaction mixture was analyzed by Western blotting with anti-Nbs1 and anti-Mre11 antibodies.

Since we found multiple cellular proteins that are targeted for E4-ORF3-mediated SUMOylation, we asked if E4-ORF3 affects SUMOylation of other Ad viral proteins. Ad5 E1B-55K oncoprotein is a well-known SUMO substrate, and SUMO-1 modification at the lysine 104 residue is required for the transforming activity of E1B-55K (24, 25). In agreement with previous reports (26, 27), E1B-55K was recruited into E4-ORF3 tracks in HeLa cells infected with an E4-ORF6-deficient Ad5 mutant virus (*dl*355), while it colocalized with viral replication centers in the absence of E4-ORF3 and E4-ORF6 (*dl*355-*in*ORF3) at 12 hpi (Fig. 3A). Next, to determine SUMOylation levels of E1B-55K, His_6_-tagged SUMO-expressing HeLa cells were infected with *dl*355 or *dl*355-*in*ORF3 for 12 h. Since the stable HeLa cell line expressing His_6_-SUMO-1 exhibited lower Ad infectivity than did the stable His_6_-SUMO-3 HeLa cell line, we infected cells with titers indicated in Fig. 3 to obtain similar levels of viral protein expression. Consistent with previous reports, E1B-55K was modified by both SUMO-1 and SUMO-3 in Ad5-infected cells (Fig. 3B). Surprisingly, only SUMO-1, but not SUMO-3, conjugation to E1B-55K was significantly increased by E4-ORF3 (Fig. 3B), indicating that E4-ORF3 enhances SUMO-1 conjugation to E1B-55K in the absence of E4-ORF6.

To further investigate the effect of E4-ORF3 on E1B-55K SUMOylation using an *in vitro* assay, Ad5 E1B-55K was purified from E. coli (GST-E1B-55K). Using the *in vitro* SUMO reaction, a single SUMO-1-conjugated Ad5 E1B-55K was observed, consistent with previous reports (24, 25). Interestingly, this SUMO-1 modification was enhanced by incubation with Ad5 E4-ORF3 (Fig. 3C), even though the increase in SUMOylation by E4-ORF3 was less than that observed with TIF-1γ and TFII-I (Fig. 2). Collectively, these data suggest that E4-ORF3 enhances SUMO-1 conjugation to E1B-55K by E4-ORF3 E3 ligase activity as well as recruitment of E1B-55K to its nuclear inclusions. These results provide the first direct evidence that E4-ORF3 functions as a SUMO E3 ligase for a viral protein as well as cellular proteins.

Next, we determined if E4-ORF3 directly functions as a SUMO E3 ligase for Nbs1 and Mre11, which were the first identified SUMO targets affected by E4-ORF3 in cultured cells (17). Nbs1 and Mre11were purified from E. coli (GST-Nbs1 and GST-Mre11) and their SUMOylation levels assessed by incubating with Ad5 E4-ORF3 in *in vitro* reactions. SUMO-1 and SUMO-3 conjugation to Nbs1 occurred weakly in the absence of E4-ORF3, but the modifications were significantly increased by E4-ORF3 (Fig. 3D, top). In the case of Mre11, no SUMO-modified Mre11 was detected in the absence of E4-ORF3, but weak SUMO-1 modification and pronounced SUMO-3 modification were observed in the presence of E4-ORF3 (Fig. 3D, bottom). These results demonstrate that Ad5 E4-ORF3 indeed functions as a SUMO E3 ligase of Mre11 and Nbs1. Although Ad5 E4-ORF3 still exhibited E3 ligase activity on all of the substrates examined, E4-ORF3-induced SUMOylation of Mre11, Nbs1, and E1B-55K was much weaker than that of TIF-1γ and TFII-I. All of these substrates were purified under the same native (nondenaturing) conditions, implying the possible involvement of cellular factors for SUMOylation *in vivo*.

### E4-ORF3-mediated relocalization of TIF-1γ into nuclear inclusions does not require SUMO conjugation.

**FIG 4:**
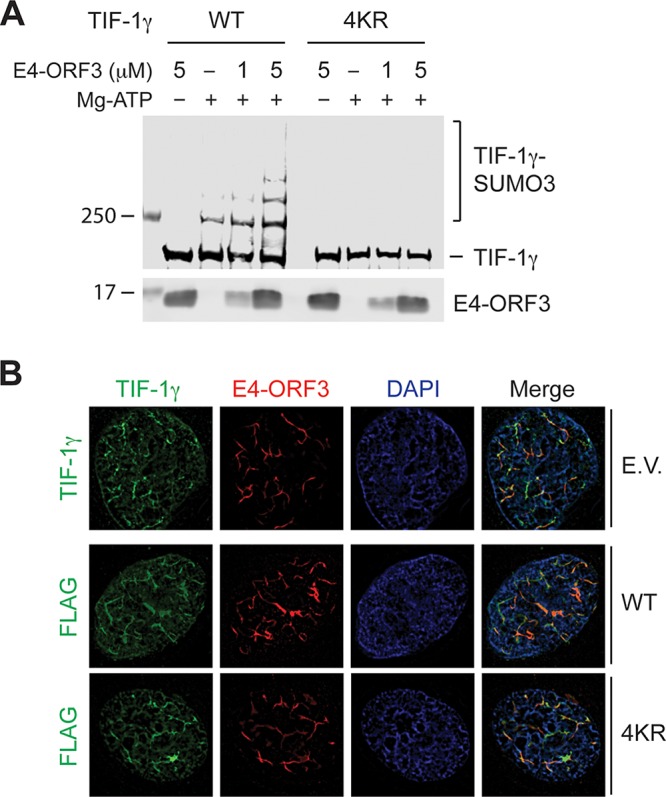
E4-ORF3-mediated TIF-1γ relocalization is independent of SUMO conjugation. (A) GST-TIF-1γΔC (wild type [WT]) or a SUMO-deficient mutant (4KR) with the indicated concentrations of Ad5 E4-ORF were incubated in the SUMO-3/E1/E2 reaction mixture described in Fig. 1D. The levels of SUMO conjugation were determined by Western blotting with the anti-TIF-1γ antibody. (B) HBEC3-KT cells transfected with HA-E4-ORF3 along with empty vector (E.V.), FLAG-TIF-1γ WT, or 4KR plasmid were immunostained with the anti-E4-ORF3 antibody and anti-TIF-1γ (top) or anti-FLAG antibody (bottom).

In our previous study, suppression of global SUMO conjugations by expressing SUMO-specific protease (SENP) 1 did not affect sequestration of Mre11 and Nbs1 into E4-ORF3 nuclear inclusions (17), suggesting that SUMOylation of E4-ORF3 target proteins may not be required for relocalization to occur. Since SUMOylation sites of TIF-1γ have been identified at lysines 776, 793, 796, and 839 (28), we examined the effect of TIF-1γ SUMOylation on subcellular relocalization by E4-ORF3 using this SUMO-deficient mutant, 4KR (28). Full-length TIF-1γ wild-type and 4KR were purified from E. coli (GST-TIF-1γ-WT and -4KR), and an *in vitro* SUMOylation assay was performed. As expected, the TIF-1γ 4KR mutations resulted in a complete failure of SUMOylation in the absence or presence of Ad5 E4-ORF3 (Fig. 4A). Next, human bronchial epithelial cells (HBEC-3KT) were cotransfected with an HA-E4-ORF3 expression vector and empty, FLAG-TIF-1γ-WT, or -4KR expression plasmids and TIF-1γ subcellular localization determined by immunofluorescence. Consistent with previous reports, both endogenous and transiently expressed TIF-1γ were relocalized by E4-ORF3 (Fig. 4B, E.V. and WT, respectively). TIF-1γ relocalization was observed in all cells that expressed E4-ORF3. Interestingly, the SUMOylation-defective TIF-1γ-4KR mutant was also relocalized into E4-ORF3 nuclear inclusions (Fig. 4B, 4KR). These results provide compelling evidence that E4-ORF3-mediated relocalization of cellular proteins into nuclear inclusions is independent of their SUMO modification status.

### E4-ORF3 interacts with UBC9 in the presence of SUMO-3.

**FIG 5:**
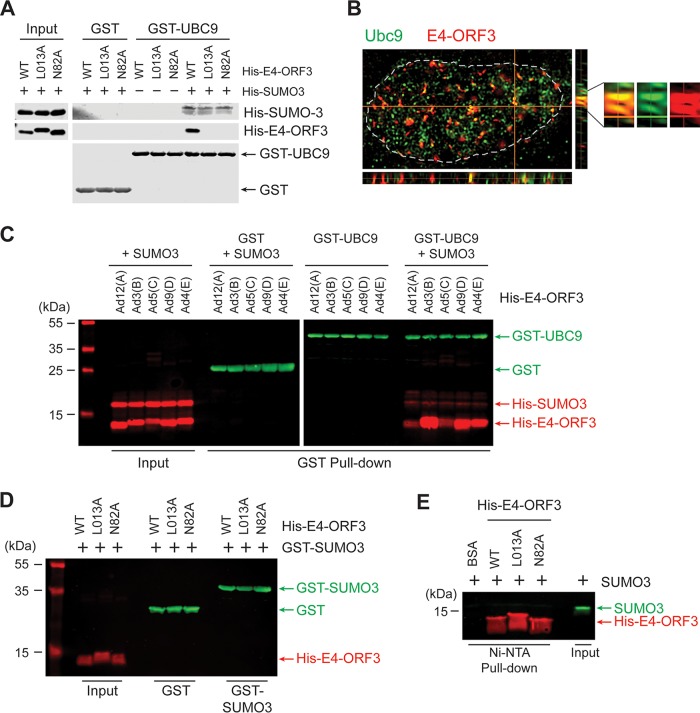
Interactions between SUMO, UBC9, and Ad E4-ORF3. (A) His_6_-E4-ORF3-WT, -L103A, or -N82A alone or together with His_6_-SUMO-3 was incubated with glutathione agarose-bound GST or GST-UBC9. Input and GST pulldown products were examined by Western blotting using anti-GST and anti-His antibodies. (B) HeLa cells transfected with HA-E4-ORF3 plasmid were fixed and immunostained with anti-E4-ORF3 and anti-UBC9 antibodies. Images were captured using structured illumination microscopy (SIM). *x*-*z* and *y*-*z* section images at the orange lines are shown at the bottom and right, respectively. Enlarged section images of the indicating region are shown. (C) GST pulldown was performed the same way as described for panel A using E4-ORF3 from Ad12, 3, 5, 9, and 4. (D) His_6_-E4-ORF3-WT or -L103A or -N82A mutant proteins were incubated with GST or GST-UBC9. (E) His_6_-E4-ORF3-WT or -L103A or -N82A mutant proteins were incubated with untagged SUMO-3. Ni^2+^-NTA agarose pulldown and input were analyzed by Western blotting with anti-His and anti-SUMO-2/3 antibodies.

We showed binding of Ad5 E4-ORF3 with UBC9 in cultured cells by coimmunoprecipitation (20). To assess whether this binding is direct or requires other factors, GST pulldown analyses were carried out using recombinant proteins, GST and GST-UBC9, and His_6_-tagged proteins E4-ORF3-WT, -L103A, and -N82A. Point mutations at the highly conserved L103 or N82 residues in E4-ORF3 still allow the formation of an E4-ORF3 dimer but completely block polymer assembly (29, 30). Surprisingly, Ad5 E4-ORF3 was not coprecipitated with UBC9 alone (Fig. 5A), indicating that they are not directly associated each other. Strikingly, the addition of SUMO-3 to the binding mixture enabled efficient E4-ORF3-WT pulldown with UBC9 (Fig. 5A). In contrast, the L103A and N82A mutant proteins were completely defective in this binding (Fig. 5A), suggesting that E4-ORF3 polymerization is crucial for this interaction to occur.

In our previous study, we demonstrated that mutations in the predicted SUMO-interacting motif (SIM) of E4-ORF3 (amino acids 103 to 106) did not affect E4-ORF3-mediated TIF-1γ SUMOylation (20). To further test the interaction between E4-ORF3 and SUMO-3, we incubated His_6_-E4-ORF3-WT and L103A and N82A mutant proteins, with either GST-SUMO-3 or untagged SUMO-3, followed by pulldown with glutathione agarose or nickel ion-nitrilotriacetic acid (Ni^2+^-NTA) agarose beads (Fig. 5D and E). There was no detectable coprecipitation of E4-ORF3 and SUMO-3 with either approach, indicating that E4-ORF3 does not bind SUMO-3 alone. These results establish that UBC9, SUMO-3, and E4-ORF3 proteins form a stable ternary complex.

Since E4-ORF3 proteins from five different Ad types were found to possess SUMO E3 ligase activities, we next questioned whether the ternary interactions of E4-ORF3, SUMO-3, and UBC9 are conserved among Ad types. We confirmed that E4-ORF3 proteins from Ad 12, 3, 9, and 4 all bound SUMO-3/UBC9 (Fig. 5C), demonstrating that the ability of E4-ORF3 to form ternary interactions with SUMO and UBC9 is conserved across Ad species.

Previous reports have shown that E4-ORF3 recruits SUMO-1 and -2/3 into the nuclear inclusions (7, 17). To determine the subcellular localization of UBC9 in the presence of E4-ORF3, E4-ORF3 was expressed in HeLa cells, and endogenous UBC9 localization was analyzed by high-resolution microscopy. UBC9 mostly localized within the nucleus in a diffuse pattern, and E4-ORF3 expression did not make significant changes in this localization. However, colocalization of UBC9 and E4-ORF3 was observed at multiple sites throughout the nucleus in all cells that expressed E4-ORF3 (Fig. 5B).

### E4-ORF3 associates with backside SUMO-bound UBC9.

**FIG 6:**
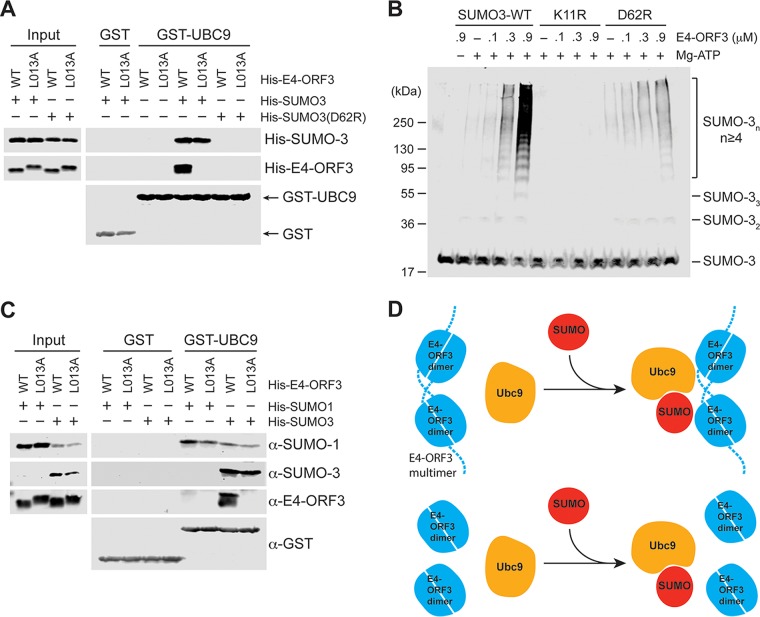
E4-ORF3 interacts with backside SUMO-bound UBC9. (A) A GST pulldown assay was performed as described in Fig. 5A using SUMO-3-WT and -D62R mutant proteins. (B) SUMO-3-WT and -K11R and -D63R mutant proteins were incubated with increasing concentrations of Ad5 E4-ORF3 (0.1, 0.3, and 0.9 μM). The levels of poly-SUMO chain formation were determined by Western blotting with anti-SUMO-2/3 antibody. (C) The GST pulldown assay was conducted using SUMO-1 and SUMO-3. The anti-SUMO-1 and anti-SUMO-2/3 antibodies were used to detect SUMO-1 and SUMO-3, respectively. The anti-SUMO-1 antibody partially cross-reacted with SUMO-3 protein. (D) The proposed model of Ad E4-ORF3 interaction with UBC9 and backside-bound SUMO.

We sought to understand the mechanism of how E4-ORF3 associates with UBC9 together with SUMO-3. The C-terminal glycine of SUMO forms a thioester linkage via the catalytic cysteine of UBC9. A second SUMO moiety may bind noncovalently on the backside of UBC9 that is opposite to the active-site cysteine 93 residue (31). For the backside binding interaction, the interfaces are found to be formed by basic amino acids in UBC9 (R13, R17, and H20) and acidic amino acids in SUMO (E67 in SUMO-1, D63 in SUMO-2, and D62 in SUMO-3) (32). Since the GST pulldown assays were conducted under conditions without E1 and ATP, SUMO conjugation to the catalytic cysteine of UBC9 did not occur, suggesting the possibility of a requirement for noncovalent binding between SUMO-3 and UBC9 for interaction with E4-ORF3. To test this possibility, the UBC9 backside-binding-deficient SUMO-3-D62R mutant was purified from E. coli, and GST pulldown assays were conducted as described above. Remarkably, E4-ORF3 was not able to interact with UBC9 in the presence of SUMO-3-D62R instead of SUMO-3-WT (Fig. 6A), indicating that backside binding by SUMO-3 to UBC9 is essential for the ternary interaction with Ad5 E4-ORF3. To examine the correlation between the E4-ORF3 SUMO E3 ligase activity and ternary interactions, *in vitro* poly-SUMOylation assays were carried out using the SUMO-3-WT, SUMO-3-D62R mutant, and polymerization-defective SUMO-3-K11R mutant in the presence of Ad5 E4-ORF3 in the absence of substrate. As expected, the SUMO-3-K11R polymerization mutant showed no SUMO conjugation. Poly-SUMOylation of the SUMO-3-D62R mutant occurred poorly in the absence of E4-ORF3, and the addition of increasing amounts of E4-ORF3 exhibited only weak changes in D62R chain formation compared to wild-type SUMO-3 (Fig. 6B). These results support the conclusion that backside binding of SUMO-3 to UBC9 is required for enhanced poly-SUMO chain formation by E4-ORF3.

SUMO-1 and SUMO-2 are found to bind UBC9 with similar affinities, although UBC9 backside binding by SUMO-2/3 is implicated in poly-SUMO chain formation (31). We asked whether E4-ORF3 has any SUMO paralog preference for the ternary interaction with UBC9 by comparing pulldown with SUMO-1 and SUMO-3. Importantly, Ad5 E4-ORF3 exhibited a very weak interaction with SUMO-1/UBC9 compared to SUMO-3/UBC9 (Fig. 6C), demonstrating that E4-ORF3 preferentially binds to SUMO-3/UBC9 over SUMO-1/UBC9.

## DISCUSSION

SUMO E3 ligases increase the rate of SUMO transfer from the E2-conjugating enzyme UBC9 to substrates by recruiting SUMO-charged UBC9 and substrates into a complex and stabilizing SUMO-charged UBC9 in an activated closed conformation (18, 19). The activities of known cellular SUMO E3 ligases fall into two categories. Members of the Siz/PIAS family of SUMO E3 ligases contain a RING domain which interacts with SUMO-charged UBC9. Siz/PIAS proteins are thought to function in an analogous manner to RING-type ubiquitin E3 ligases and function as adaptors to promote SUMO-UBC9 and substrate interaction to facilitate conjugation. RANBP2 and ZNF451 are SUMO E3 ligases that lack a RING domain. These proteins bind SUMO and SUMO-charged UBC9 and promote SUMO conjugation by serving as adaptor proteins between substrates and SUMOylation components. In this study, we investigated how the Ad E4-ORF3 protein functions as a SUMO E3 ligase and define parameters associated with substrate specificity.

The Ad5 E4-ORF3 protein has been shown to relocalize SUMO and SUMOylated substrates into nuclear inclusions to facilitate SUMO conjugation in infected cells (17). The structure of the E4-ORF3 protein has been solved (30), and E4-ORF3 does not have a RING domain. E4-ORF3 residues 103 to 107 are a predicted SIM, but these sequences are not critical for E4-ORF3 SUMO ligase activity (20). Here, we showed that the E4-ORF3 protein does not directly bind either SUMO or UBC9 alone. Instead, E4-ORF3 binds UBC9 only in a trimeric complex with backside-bound SUMO (Fig. 5 and 6). Charging of UBC9 via thioester linkage of SUMO to the catalytic cysteine residue is not required for these interactions to occur. Furthermore, our results using E4-ORF3 N82A and L103A mutants revealed that E4-ORF3 polymerization is crucial for this interaction. Based on these results, we propose a model for a trimeric interaction of E4-ORF3 with UBC9 and SUMO (Fig. 6D). This trimeric interaction could be due to a conformational change of UBC9 by SUMO backside binding. However, this possibility seems unlikely since backside binding of SUMO to UBC9 does not promote any significant change in UBC9 structure (33, 34). Rather, we think it is more likely that E4-ORF3 binds to a newly emerged interface created by UBC9 and backside-bound SUMO. We hypothesize that E4-ORF3 functions to recruit the SUMO machinery, including UBC9 (Fig. 5B), and its substrates in proximity of one another using its polyvalent scaffold (29, 30) to assemble higher-order protein complexes.

To elucidate the characteristics of E4-ORF3-mediated SUMO conjugation and conservation of function, we analyzed E4-ORF3 proteins from different Ad species and divergent substrate proteins. The *in vitro* E3 ligase activity of Ad5 E4-ORF3 was conserved across the Ad types tested (species A to E) (Fig. 1), even though Ad5 E4-ORF3 shares only 50% or less amino acid sequence identity with E4-ORF3 of other Ad types. In infected cells, SUMO conjugation to proteins occurred only when substrates were recruited into E4-ORF3 nuclear inclusions generated by each Ad type (12, 16, 17, 20). Why the E4-ORF3 proteins of different Ad species differ in their abilities to relocalize certain cellular proteins such as Nbs1, Mre11, and TFII-I (7, 12) is not understood structurally or functionally. We previously suggested that relocalization of cellular proteins by E4-ORF3 is a prerequisite for their SUMOylation (17). Since the SUMOylation-deficient TIF-1γ protein was efficiently recruited into E4-ORF3 inclusions (Fig. 4), SUMOylation of target proteins is not a requirement for E4-ORF3-mediated relocalization, confirming that relocalization is followed by SUMO conjugation. Collectively, our data demonstrate that both E4-ORF3 polymerization, which is essential for its *in vitro* SUMO E3 ligase activity, and substrate recruitment into the nuclear scaffold are required for E4-ORF3-mediated SUMOylation in infected cells.

We previously reported that E4-ORF3 promotes SUMO-2 or SUMO-3 conjugation to substrates with a higher efficiency than SUMO-1 in infected cells with Nbs1, Mre11, and, to a lesser extent, TFII-I (12, 17). Here, we found that E4-ORF3 preferentially formed a ternary interaction with SUMO-3/UBC9 compared to SUMO-1/UBC9 (Fig. 6), consistent with these results. A greater extent of SUMO-3, compared to SUMO-1, conjugation to these substrates was recapitulated *in vitro* (Fig. 2 and 3), indicating that preferential binding of E4-ORF3 to SUMO-3/UBC9 may represent the basis for these results. We also observed E4-ORF3-mediated SUMO-1 conjugation to the Ad5 E1B-55K protein *in vitro* and enhancement of E1B-55K SUMO-1, but not SUMO-3, conjugation by E4-ORF3 in infected cells (Fig. 3). This is the first demonstration of the involvement of the E4-ORF3 protein in the regulation of E1B-55K SUMOylation, a process that is critical for certain activities of this protein (24, 25). Interestingly, E4-ORF3 cooperates with the Ad E1A and E1B proteins to transform rodent cells, and the E4-ORF3 protein was found to interact with E1B-55K (35). Since SUMO-1 modification of E1B-55K is required for its transformation properties (24), it seems likely that the role of E4-ORF3 in transformation may be to function as a SUMO E3 ligase and promote E1B-55K SUMOylation.

As we and others previously reported (12, 15, 20), E4-ORF3-mediated SUMOylation results in proteasomal degradation of TFII-I and TIF-1γ but not Mre11 and Nbs1. The basis for this discrimination in the outcome of E4-ORF3-induced SUMOylation of cellular substrates in not clear at this time. In this study, we confirmed that E4-ORF3 functions as a SUMO E3 ligase of Mre11 and Nbs1, although *in vitro* SUMOylation levels of Mre11 and Nbs1 were much lower than those of TIF-1γ and TFII-I, implying a possibility for the involvement of cellular factors. The types of SUMO modification that occur with different substrates targeted by E4-ORF3, e.g., mono- versus poly-SUMOylation, may explain the different outcomes of the process. A wide range of different DNA and RNA viruses impact host protein SUMOylation (36). However, only a few viral proteins have been described as SUMO E3 ligases. Kaposi’s sarcoma-associated herpesvirus (KSHV) encodes a SUMO E3 ligase, K-bZIP, that is SIM dependent and specific for SUMO-2/3 modification (37). The Ad E1B-55K protein is a SUMO E3 ligase that is SUMO-1 specific (38). E1B-55K regulates SUMOylation by an unknown mechanism. The Ad E4-ORF3 protein functions as a SUMO E3 ligase by an entirely novel mechanism. It binds a backside-bound SUMO-UBC9 complex and tethers this complex with a number of different cellular substrates through higher-order protein-protein interactions. E4-ORF3 directs both SUMO-1 and SUMO-2/3 conjugation and regulates cellular proteins involved in a range of functions, including IFN signaling, the DDR, and transcriptional regulation.

## MATERIALS AND METHODS

### Cells, viruses, and transfection.

The human bronchial epithelial cell (HBEC) 3-KT (American Type Culture Collection [ATCC]) was cultured in airway epithelial cell basal medium (ATCC) supplemented with the bronchial epithelial cell growth kit (ATCC) and maintained according to the provider’s instructions. HeLa cells were grown in Dulbecco’s modified Eagle medium (DMEM) supplemented with 10% (vol/vol) calf serum. The HeLa cell lines stably expressing His_6_-tagged SUMO-1 and His_6_-tagged SUMO-3 were generously provided by Ronald Hay (University of Dundee); these cells were selected to express His_6_-tagged SUMO proteins at levels comparable to endogenous SUMO proteins (39). For transfection, Lipofectamine 2000 (Invitrogen, Thermo Fisher Scientific) was used for HBEC3-KT cells, and polyethylenimine (PEI; Polysciences) was used for HeLa cells. The viruses used in this study include wild-type Ad5, *dl*355 (ΔE4-ORF6), and *dl*355-*in*ORF3 (ΔE4-ORF3/ΔE4-ORF6) (40). The E1 replacement viruses that express HA-tagged E4-ORF3 of Ad3, 4, 5, 9, and 12 under the control of a cytomegalovirus (CMV) promoter were previously described (6, 41). Viral preparation and infection were performed as described previously (6).

### Plasmids and recombinant proteins.

The HA-tagged E4-ORF3 expression vector was described previously (17). FLAG-tagged TIF-1γ and SUMO-deficient mutant 4KR (K776R, K793R, K796R, and K839R) (28) expression plasmids were provided by Rita Rimokh (Université de Lyon). Recombinant human SUMO E1 and E2 enzymes and untagged SUMO-3 were purchased from Boston Biochem. The rest of the recombinant proteins used in this study were expressed in Rosetta E. coli cells and purified using either Ni^2+^-NTA agarose (Qiagen) or glutathione agarose (Sigma-Aldrich), as previously described (20, 29). The E4-ORF3 coding regions of human Ad3, 4, 5, 9, 12, and Ad5 E4-ORF3 mutants, the L103A and N82A mutants, were inserted into the bacterial expression plasmid pProEx HTb. GST-tagged TIF-1γΔCT (20) and 4KR were cloned in pGEX 4T-1, GST-tagged Nbs1 was cloned in pGEX-3X, and GST-tagged TFII-I and Mre11 were cloned in pGEX-KG. Based on advice from David A. Ornelles (Wake Forest University) about unwanted recombination between the E1B-55K coding region and E. coli chromosome DNA, we generated an E1B-55K expression plasmid containing scrambled synonymous codons while maintaining the wild-type protein sequence and optimized for protein expression in E. coli (42). The altered Ad5 E1B-55K coding sequence was kindly designed by Justin Gardin (Stony Brook University). The optimized E1B-55K coding region was synthesized from Biomatik and cloned in pGEX-KG. The GST-Ubc9 expression plasmid pGEX-3X-Ubc9 was provided by Stephen Goff (Columbia University). SUMO-1 and SUMO-3 coding regions were cloned in pET28a. SUMO-3-D62R and SUMO-3-K11R mutants were generated by site-directed mutagenesis.

### Antibodies.

The anti-TIF-1γ rabbit antibody was generated as previously described (13). The anti-E4-ORF3 rat monoclonal antibody (6A-11) was provided by Thomas Dobner (Heinrich-Pette Institute), the anti-E1B-55K (2A6) mouse monoclonal antibody was from Arnold Levine (Princeton University), and the rabbit polyclonal anti-DBP antibody was from Peter van der Vliet (University of Utrecht). The mouse anti-RanGAP1, rabbit anti-TFII-I, and rabbit anti-GST antibodies were purchased from Santa Cruz Biotechnology. The anti-Mrell and anti-Nbs1 mouse monoclonal antibodies were from GeneTex. The rabbit anti-SUMO-1 antibody was from Cell Signaling Technology, and the anti-Ubc9 rabbit polyclonal antibodies were from Cell Signaling Technology and Proteintech. The anti-SUMO-2/3 rabbit polyclonal antibody was from Thermo Fisher Scientific, the anti-His mouse monoclonal antibody was from Clontech, and the anti-HA rabbit polyclonal antibody was from Rockland.

### *In vivo* SUMOylation assay.

The parent HeLa or His_6_-tagged SUMO-1- and SUMO-3-expressing HeLa cells were infected with viruses indicated in the text, and SUMO conjugates were captured and analyzed as described by Tatham et al. (39).

### *In vitro* SUMOylation assay.

SUMO conjugation reactions were performed using 100 nM substrate protein, 50 μM SUMO, 20 to 50 nM E1, 150 to 250 nM E2, and 0.1 to 9 μM E4-ORF3 in a SUMOylation reaction buffer (20 mM HEPES [pH 7.5], 0.5 mM EGTA, 1 mM dithiothreitol [DTT], 0.5% Tween 20, 2 mM ATP, and 5 mM MgCl_2_) at 37°C for the indicated time periods. Poly-SUMO-3 chain assembly reactions were performed as described above minus substrate protein.

### GST pulldown.

The following amounts of recombinant proteins were used for pulldown assays: 0.1 nM GST, 0.1 nM GST-Ubc9, 0.25 nM His_6_-E4-ORF3, and 0.4 nM His_6_-SUMO. His_6_-E4-ORF3 and His_6_-SUMO proteins in binding buffer (10 mM Tris [pH 8.0], 2.5 mM β-mercaptoethanol, and 0.1% Triton X-100) were precleared by incubating with glutathione agarose beads at 4°C for 30 min. GST and GST-Ubc9 proteins were incubated with glutathione agarose beads at 4°C for 60 min in binding buffer. Precleared E4-ORF3 and SUMO proteins were added into the binding mix and incubated for an additional 3 h. The beads were washed five times with wash buffer (10 mM Tris [pH 8.0], 2.5 mM β-mercaptoethanol, and 0.5% Triton X-100). Bead-bound proteins were eluted in 2× Laemmli sample buffer and analyzed by Western blotting.

### Immunofluorescence assay and microscopy.

Cells were fixed with 4% (vol/vol) formaldehyde and permeabilized with 0.5% Triton X-100. After blocking in 10% goat serum, HA-tagged E4-ORF3 from Ad3, 4, 5, 9, and 12, and endogenous TIF-1γ were detected with anti-HA and anti-TIF-1γ antibodies, respectively. Cell images were acquired on an Axiovert 200M digital deconvolution microscope (Zeiss) and analyzed using the AxioVision software. Subcellular localization of TIF-1γ wild-type and SUMO-deficient 4KR, E1B-55K, and endogenous Ubc9 was determined by structured illumination microscopy (N-SIM; Nikon), and images were reconstructed and analyzed using the NIS-Elements software (Nikon).
